# Bacterial Contamination in Total Joint Replacement: Should We Change into a New Set of Clean Scrub Suits Whenever Entering the Operating Room?

**DOI:** 10.3390/life13071615

**Published:** 2023-07-24

**Authors:** Toshiyuki Tateiwa, Yasuhito Takahashi, Tsunehito Ishida, Toshinori Masaoka, Takaaki Shishido, Kengo Yamamoto

**Affiliations:** 1 Department of Orthopedic Surgery, Tokyo Medical University, Tokyo 160-0023, Japan; yasuhito@tokyo-med.ac.jp (Y.T.); tsune725@aol.com (T.I.); tmumasaoka@nifty.com (T.M.); tstmu@tokyo-med.ac.jp (T.S.); kyamamoto@pf6.so-net.ne.jp (K.Y.); 2 Department of Bone and Joint Biomaterial Research, Tokyo Medical University, Tokyo 160-0023, Japan

**Keywords:** bacterial contamination, periprosthetic joint infection (PJI), total joint replacement (TJR), scrub suit, coagulase-negative staphylococci (CNS)

## Abstract

Background: The aims of this study are as follows: (1) to investigate the level and probability of bacterial contamination on scrub suits over time for medical personnel working inside and/or outside the operating room (OR) area; (2) to discuss the protective role of cover gowns against bacterial contamination; and (3) to consider the necessity of changing into clean suits whenever entering the OR in terms of preventing periprosthetic joint infection (PJI) in total joint replacement (TJR). Methods: The bacterial colony count was examined on the chest area of the scrub suits worn during various daily clinical practices. The genus/species of the contaminants were identified by matrix-assisted laser deposition ionization-time of flight mass spectrometry (MALDI-TOF MS). Results: The scrub suits worn outside the OR area showed a greater level of bacterial contamination than those worn inside the OR area, although the colony counts tended to increase over time both in and out of the area. The probability of contamination involving coagulase-negative staphylococci (CNS) (known as the primary cause of PJI) did not increase significantly in the scrubs worn inside the OR area, but did increase significantly after a long-time departure from the OR area. Conclusions: Our results suggest that wearing scrub suits outside and/or the long duration of wearing the same suits can significantly increase the level and probability of any bacterial contamination (including CNS) on scrub suits. We also found that wearing a cover gown over scrub suits outside the OR area may have only a limited protective role against bacterial contamination.

## 1. Introduction

In total joint replacement (TJR) surgery, the contamination level of operating room (OR) personnel and their movement may affect the incidence of surgical site infection (SSI) and periprosthetic joint infection (PJI) through the dispersal of microorganisms into the OR environment [1]. As the guidelines of the National Institute for Health and Care Excellence (NICE) recommend, “staff wearing non-sterile theater wear should keep their movements in and out of the OR area to a minimum” [2]. Many orthopedic surgeons anecdotally believe that the contamination of OR attire can be a considerable risk factor associated with SSIs/PJIs. At the Second International Consensus Meeting on Musculoskeletal Infection (ICM), in response to the question “Does strict adherence to not wearing OR attire outside the hospital or outside the restricted OR area reduce the risk of SSIs/PJIs?”, a strong consensus exists that “we recommend that OR personnel wearing attire that has come into contact with areas outside the restricted OR environment not wear the same attire during elective arthroplasty or complex orthopedic procedures” [3]. 

Nevertheless, in reality, many surgical staff continue to wear the same OR attire (often referred to as surgical scrub suits) even upon re-entry to the OR after a departure from the restricted OR environment to the outside. Their common behavior is leaving the OR area wearing their suits or white coats (cover gowns) over their scrub suits and subsequently returning to the OR without changing into clean scrub suits. The American College of Surgeons (ACS) guidelines recommended that “OR scrubs should not be worn in the hospital facility outside of the OR area without a clean lab coat or appropriate cover up over them” [4]. However, there has been no clear evidence to support the protective role of the cover gown against bacterial contamination. Also, there is little or no research to demonstrate that wearing a scrub suit outside the OR and returning without changing into a new suit can be a potential risk factor for SSIs/PJIs [5,6]. Especially in an era of time and cost containment, the necessity of changing into new suits whenever entering the OR is still a matter of controversial discussion due to a lack of scientific evidence. 

In this study, we hypothesized that there would be a difference in the level of the bacterial contamination of surgical scrub suits depending on whether they are worn inside or outside the OR area. The aims of the present study are as follows: (1) to investigate the level and probability of bacterial contamination on scrub suits over time for medical personnel working inside and/or outside the OR area; (2) to discuss the protective role of cover gowns against bacterial contamination; and (3) to consider the necessity of changing into a new set of clean suits whenever entering the OR in terms of SSI/PJI prevention in TJR.

## 2. Methods

Approval from the Institutional Review Board (IRB) was not required for data capturing because this study focuses on investigating the bacterial contamination of surgical attire and does not involve the investigation of human or animal subjects. Twenty-six male subjects from the department of orthopedic surgery engaged in routine clinical activities inside and outside the OR area volunteered for the study. All participants were briefed about the procedures, but not informed of the research objective and sampling method.

### 2.1. Samples

In terms of the number of bacterial colonies and their genus/species identification, we evaluated the contamination level of hospital-provided surgical scrub suits (Folk Co., Ltd., Tokyo, Japan) worn by medical personnel working inside and/or outside the OR area (*n* = 65) in comparison with that of laundered unworn suits (*n* = 10) as the baseline control. The participants invariably wore hospital-provided buttoned-up white coats (Watakyu Seimoa Co., Ltd., Tokyo, Japan) as cover gowns over their scrub suits during a departure from the restricted OR environment. All scrubs and white coats were stored in a surgical locker room within the restricted OR area, which had been routinely washed by the laundry facility, but not autoclaved and wrapped separately in sterile packs. Sterile surgical space suits (Stryker Instruments, Kalamazoo, MI, USA) were also worn over the scrub suits as cover gowns during total hip and knee arthroplasty (THA and TKA), and were undressed immediately after surgery for sampling. None of the participants wore ‘chest accessories’ such as stethoscopes and lanyards.

The following scrub samples worn for a relatively short, middle, or long time were inspected during daily clinical practice: (1) scrub suits worn only inside the OR area under sterile space suits while performing either short-time primary THA or TKA (*n* = 12); (2) scrub suits worn only inside the OR area with no cover gowns for a middle time (without performing any surgery) (*n* = 12); (3) scrub suits worn only inside the OR area with no cover gowns for a long time (without performing any surgery) (*n* = 11); (4) scrub suits worn under white coats for short visits out of the OR area to the departmental office and/or cafeteria after performing short-time primary THA or TKA (*n* = 20); and (5) scrub suits worn only outside the OR area under white coats during overnight shift work (*n* = 10).

Based on the workplaces (in/outside the OR area) and duration (short/middle/long) of wearing the same scrubs with/without the use of cover gowns (space suits/white coats), the above scrub groups (1)–(5) were henceforth referred to as “IN_(sp)__ST”, “IN_MID”, “IN_LO”, “IN_(sp)_ + OUT_(w)__ST”, and “OUT_(w)__LO”, respectively, where IN and OUT represent scrubs worn “in” and “out” of the OR area, and ST, MID, and LO represent scrubs worn for a “short”, “middle”, and “long” duration of wearing the same suits, and the subscript (sp) and (w) represent the “space suit” and “white coat”, respectively. No subscript represents wearing the scrubs alone without the use of any cover gowns.

### 2.2. Bacterial Contamination Analysis

The bacterial colony counts of each scrub group were expressed as colony-forming units (CFU). The data sampling was conducted by pressing a contact plate (SCD-LP agar medium, Enbio Co., Ltd., Tokyo, Japan) against the chest area within ~5.5 cm fabric circle of each scrub group. All sampling was carried out by one researcher (TT). The obtained specimen was then cultured on the agar medium at 35 °C for 24 h and stored at room temperature for 24 h, after which the bacterial CFU were counted. 

In addition, to identify the genus/species of contaminants, matrix-assisted laser deposition ionization-time of flight mass spectrometry (MALDI-TOF MS) was performed on an Autoflex II TOF/TOF mass spectrometer (Bruker Daltonics GmbH, Bremen, Germany) equipped with a 337-nm pulsed nitrogen laser. The mass spectra ranging from the mass-to-charge ratio (*m*/*z*) 2000 to 20,000 were analyzed using Biotyper software (version 2.0; Bruker Daltonics) and were assigned with the MALDI Biotyper reference database library using the integrated pattern-matching algorithm of the software. The whole process from MLDI-TOF MS measurement to identification was performed automatically without any user intervention. The results of the pattern-matching process were expressed with scores ranging from 0 to 3; a score of <1.7 was considered an unreliable indication, a score of ≥ 1.7 was considered an identification of genus, and a score of ≥ 2.0 was considered an identification of species [7]. Based on the results of MLDI-TOF MS, we evaluated the probability of scrub contamination associated with any bacteria and coagulase-negative staphylococci (CNS). Note that CNS has been reported as the primary cause of PJI for THA and TKA in most reports from Europe [8,9,10,11] and Asia [12].

### 2.3. Statistical Analysis

The bacterial colony counts in the worn scrubs were compared statistically with those in the unworn controls using the Mann–Whitney U test. The chi-squared test was also applied to assess the statistical significances in the probability of scrub contamination associated with any bacteria and CNS, as compared to the control group. All statistical analyses were performed using Graphpad Prism software, version 8.3.0 (GraphPad software, Inc., San Diego, CA, USA). The statistical differences were considered significant at the *p* < 0.05 level. The *r* and *φ* were also reported as indicators of effect sizes for the Mann–Whitney U and chi-squared test results, respectively, considering that *r* and *φ* = 0.10 represent a small effect, *r* and *φ* = 0.30 represent a medium effect, and *r* and *φ* = 0.5 represent a large effect [13,14].

## 3. Results

All participants completed the study, yielding a total of 75 specimens, all of which were included in the analysis. Their median (interquartile, IQR) duration of wearing the same scrub suits was 0 (0–0) hours in the control group, 2 (2–2) hours in the IN_(sp)__ST group, 4 (3.5–4.6) hours in the IN_MID group, 8 (8–8.5) hours in the IN_LO group, 3 (2.5–4) hours in the IN_(sp)_ + OUT_(w)__ST, and 14.5 (14–14.5) hours in the OUT_(w)__LO group (Table 1).

**Figure 1:**
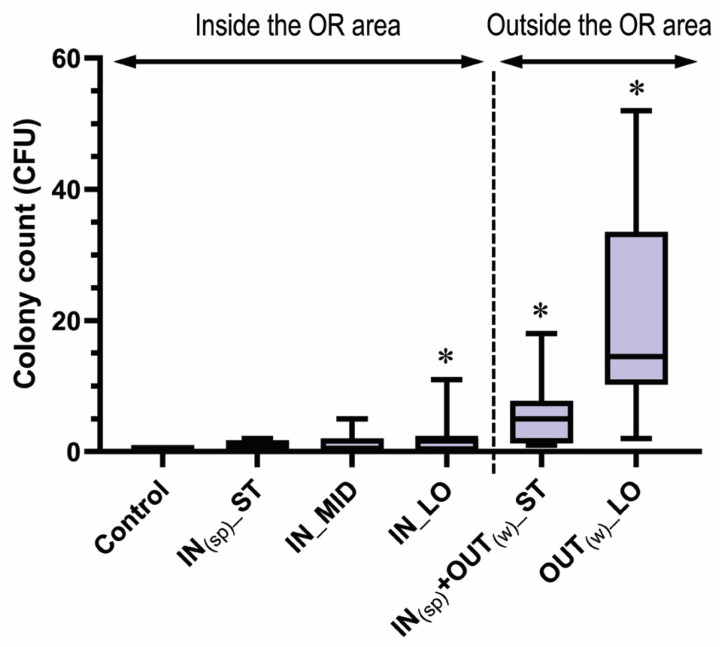
Box plots showing the bacterial colony count on the chest area of scrub suits in each group. The asterisk indicates *p* < 0.05 in comparison with the control group on the Mann–Whitney U test.

The bacterial colony count ranged from 0 to 52 CFU of the 75 specimens. Their median (IQR) bacterial colony count was 0.0 (0.0–1.0) CFU in the control, 1.0 (0.0–1.3) CFU in the IN_(sp)__ST, 0.5 (0.0–2.0) CFU in the IN_MID, 2.0 (0.3–2.0) CFU in the IN_LO, 5.0 (1.8–7.3) CFU in the IN_(sp)_ + OUT_(w)__ST, and 14.5 (11.3–29.8) CFU in the OUT_(w)__LO groups (Figure 1). According to the Mann–Whitney U test, a significantly greater increase in the colony count was detected in the IN_LO (*p* = 0.019, *r* = 0.51), IN_(sp)_ + OUT_(w)__ST (*p* < 0.0001, *r* = 0.73), and OUT_(w)__LO group (*p* < 0.0001, *r* = 0.85) than the control group, while no significant contamination was noted in the IN_(sp)__ST (*p* = 0.142, *r* = 0.35) and IN_MID group (*p* = 0.348, *r* = 0.21) (Table 2).

**Figure 2:**
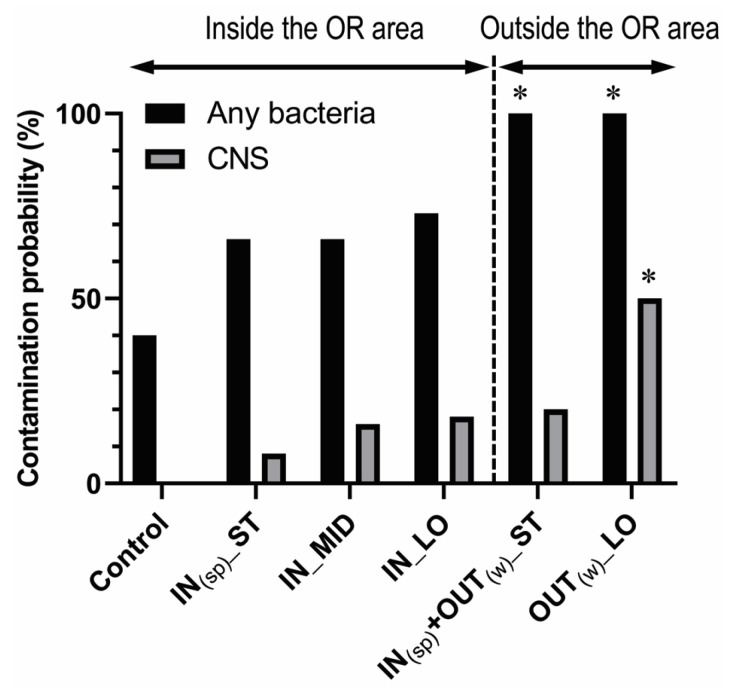
Graph showing the probability of contamination associated with any bacteria and CNS on the chest area of scrub suits in each group. The asterisk indicates *p* < 0.05 in comparison with the control group on the chi-squared test.

MALDI-TOF MS revealed that the genus *Bacillus* was predominant among all the contaminants detected in each group (Table 3). The probability of any bacterial contamination was 40% (4/10) in the control group, 66.7% (8/12) in the IN_(sp)__ST group, 66.7% (8/12) in the IN_MID group, 72.7% (8/11) in the IN_LO group, 100% (20/20) in the IN_(sp)_ + OUT_(w)__ST group, and 100% (10/10) in the OUT_(w)__LO group (Figure 2). According to the chi-squared test, a significantly higher contamination probability over the control group was found in the scrubs worn outside the OR area (IN_(sp)_ + OUT_(w)__ST [*p* = 0.0001, *φ* = 0.71] and OUT_(w)__LO [*p* = 0.003, *φ* = 0.66]). The increase in the contamination probability was found numerically but statistically insignificant in the scrubs worn only inside the OR area, i.e., IN_(sp)__ST (*p* = 0.211, *φ* = 0.27), IN_MID (*p* = 0.211, *φ* = 0.27), and IN_LO (*p* = 0.130, *φ* = 0.32) (Table 4).

CNS was detected in 0% (0/10) in the control group, 8.3% (1/12) in the IN_(sp)__ST group, 16.7% (2/12) in the IN_MID group, 18.2% (2/11) in the IN_LO group, 20% (4/20) in the IN_(sp)_ + OUT_(w)__ST group, and 50% (5/10) in the OUT_(w)__LO group (Figure 2). According to the chi-squared test, significantly higher CNS rates over the control group were noted in the OUT_(w)__LO group (*p* = 0.010, *φ* = 0.58) and not in the IN_(sp)__ST (*p* = 0.350, *φ* = 0.20), IN_MID (*p* = 0.176, *φ* = 0.29, IN_LO (*p* = 0.156, *φ* = 0.31), and IN_(sp)_ + OUT_(w)__ST (*p* = 0.129, *φ* = 0.28) groups (Table 4).

## 4. Discussion

Human skin is colonized with bacteria that are continuously shed from our skin and dispersed into the air [15,16,17,18]. Based on a strong consensus on the association between airborne bacteria and SSI, the 2013 ICM on PJI stated that “airborne particulate bacteria are a major source of contamination in the OR environment and that bacteria shed by personnel are the predominant source of these particles” [19]. In ICM, there is also a consensus that human movement within the OR should be minimized, as increased airborne dust counts are predisposed to PJI [2]. We previously demonstrated that a simple stepping motion with a contaminated scrub may dissatisfy the class 100 for ambient clean air levels in the OR environment due to significant increases in airborne particles [20]. These data potentially tell us that when only one staff member wearing a contaminated scrub is present, we cannot guarantee the required intraoperative cleanliness of the OR environment. One recent case report [21] identified a nurse anesthetist wearing a contaminated scrub as the source of the wound infection in three patients. 

Our current study demonstrated that wearing scrub suits outside and/or the long duration of wearing the same suits can result in a significant increase in the bacterial colony counts on scrub suits (Figure 1). In addition, regardless of the wearing duration, the probability of any bacterial contamination was significantly higher only in the scrubs worn outside the OR area than the control (Figure 2). In the outside environment, the concentration of airborne bacteria tends to be higher than inside a positively pressurized OR, which could potentially increase the risk of contaminating scrubs during routine activities. Furthermore, it is speculated that the greater scrub contamination outside the OR may be attributed to the frequent need for contact with patients or medical staff without wearing clean attire. In this context, re-entering the OR without changing into laundered suits can be a potential risk factor for increasing the bacterial contamination of the OR environment. However, it is noteworthy that a significantly higher probability of CNS contamination was noted only in the OUT_(w)__LO group and not in the IN_(sp)_ + OUT_(w)__ST group or the control (Figure 2). Thus, whether changing scrubs should be mandated or not in the case of the re-entry of staff after their brief visits outside while wearing cover gowns remains controversial. Nevertheless, since a lack of statistical significance does not equal to a lack of clinical relevance, further studies are needed to clarify the potential impact of such a case on the incidence of SSIs/PJIs. 

One similar study performed by Hee et al. [18] showed that brief visits out of the OR to surgical wards and nearby departmental offices did not significantly increase contamination on the chest, waist, and hip of the scrub suits. On the other hand, Sivanandan et al. [22] showed greater contamination on the scrubs worn outside the OR than those inside the OR at 2 h, but no significant difference in the amount of contamination at 4, 6, and 8 h. Their sampling sites were the back at the mid-thoracic region and the sacral region. Both studies showed that bacterial counts increased over time both in and out of the OR area, but did not identify the genus/species of the contaminants. Despite the differences in methodology, we also found a slight increase in the bacterial counts on the scrubs worn inside the OR area over time (Figure 1), but did not find a significant increase in the probability of CNS as well as any bacterial contamination up to the median 8 h (Figure 2). Therefore, wearing scrub suits inside until ~8 h is unlikely to significantly affect the incidence of SSIs/PJIs, but a longer wearing duration would potentially lead to significant contamination, even within the OR area.

So far, many guidelines recommend changing scrubs upon exit and/or re-entry to the OR [23,24,25]. The National Association of Theater Nurses (NATN) guidance recommends that staff should change into outer clothes when leaving the theater area and put on a new set when returning [23]. The Association of Perioperative Registered Nurses (AORN) recommends removing surgical attire before leaving the health care facility [24]. At the French Society of Anesthesia and Intensive Care Medicine (SFAR) and the French Society of Hospital Hygiene (SF2H), the experts suggest that “Staff not leave the operating theatre with their operating theatre attire, the objective being to limit contamination and thereby to prevent infections risk for the patients.”, “If staff must exceptionally and for imperative reasons leave the operating theatre with their operating theatre attire, they must change their clothes on their return to the operating theatre to limit infectious risk for the patient.”, and “In the event of a brief departure (a few minutes) from the operating theatre, an alternative strategy can consist in covering the operating theatre attire with a closed gown if they stay outside” [25]. 

Nevertheless, due to a lack of universal consensus, many institutions currently allow the use of scrub suits outside the OR area, and staff are not mandated to change into a new set of clean scrubs when returning. Based on a questionnaire survey of Israeli surgeons and anesthesiologists, Weinbroum et al. [26] reported that most (80%) left the OR area still wearing OR attire, 82% did not change into regular clothes later on, 63% responded that wearing covering apparel or a laboratory coat is acceptable, and 38% considered it obligatory to change to regular attire when leaving the OR. Although many recommend the use of covering the scrubs outside, the protective role of cover gowns against bacterial contamination is still unclear. McHugh et al. [27] reviewed 50 years of reports up to 2013 and concluded that theater staff should wear a single-use cover gown or coat, which should be discarded when returning to the theater complex. On the other hand, Kaplan [28] compared the degree of the contamination of scrubs between with and without cover gowns and concluded that there was no evidence of the benefit of covering gowns in maintaining the sterility of surgical scrub suits. The Japanese Association for Operative Medicine (JAOM) stated that the usefulness of wearing a cover gown over the surgical gown when leaving the OR is an unresolved issue [29]. In practice, staff do not always wear a clean cover gown over the scrub suit when leaving the OR. It is noteworthy that the IN_(sp)_ + OUT_(w)__ST group using cover gowns showed greater colony counts (*p* = 0.002, *r* = 0.56) and contamination probability (*p* = 0.006, *φ* = 0.49) than the IN_MID group with no cover gowns and a longer wearing time, suggesting that wearing a clean buttoned-up cover gown outside the OR seems unlikely to provide effective protection against bacterial contamination. Also, these comparisons imply that the outside environment might contaminate scrubs faster than the inside. It is conjectured that the cover gown, unlike surgical space suits, is not hermetically sealed against contaminants with positive pressure, which could potentially allow some level of bacterial penetration through openings such as the collar and cuffs.

Our study has some limitations. First, the number of samples analyzed was relatively small. However, medium-to-large effect sizes were obtained for all significant comparisons, indicating that a larger sample size would not be required to detect these differences. Second, male subjects are known to disperse significantly more normal skin microorganisms, and notably more *Staphylococcus aureus*, than female subjects [30,31]. Therefore, the bacterial counts might potentially be reduced in female subjects, although we only included male subjects in this study. Third, the sampling site was limited to the chest area of the scrub suits above the height of the patient on an operating table. Hee et al. [18] showed that the mean bacterial counts were lower in the chest compared to the hip and waist areas. Therefore, our data may lead to an underestimation of a contamination risk for the OR environment. Fourth, the OUT_(w)__LO group showed a relatively high degree of variability in the colony count, which could be due to variations in the tasks involved and workplaces outside the OR. We speculate that greater patient contact may lead to greater contamination on the chest during overnight shift work. Lastly, it is unclear what level of bacterial contamination is considered clinically significant in terms of SSIs/PJIs.

Despite these limitations, our results suggest that wearing scrub suits outside and/or the long duration of wearing the same suits can result in a significant increase in the level and probability of any bacterial contamination (including CNS) on scrub suits. Although the bacterial colony counts would increase over time both in and out of the OR area, the outside is likely to contaminate scrubs faster than the inside. In addition, wearing a cover gown outside the OR may have only a limited role in protecting against contamination. Based on the current results, we recommend that, after a long-time departure from the OR area wearing the same suits, surgical staff should change into a new set due to an increased probability of CNS contamination. On the other hand, it would be controversial that a brief departure from the OR area would require a clean buttoned-up cover grown over a scrub suit within one or two hours, since it did not significantly increase the probability of CNS contamination. Nevertheless, since a lack of statistical significance does not equal to a lack of clinical relevance, further studies are needed to clarify the potential impact on the incidence of SSIs/PJIs. We believe that promoting a shared understanding and adoption of the importance of changing into clean scrubs upon re-entering the OR among all hospital personnel, including anesthetists and other non-surgeons, can be an effective strategy to minimize the risk of bacterial contamination during total joint replacement surgeries. In the future, there will be a need to verify the reduction in SSIs/PJIs and assess its cost-effectiveness through the practice of changing into clean scrubs.

## Figures and Tables

**Table 1:** Duration of wearing the same scrub suits in each group. Values are median (interquartile range, IQR).

Group	The Duration of Wearing the Same Suit (Hours), Median (IQR)
Control	0.0 (0.0 to 0.0)
IN_(sp)__ST	2.0 (2.0 to 2.0)
IN_MID	4.0 (3.5 to 4.6)
IN_LO	8.0 (8.0 to 8.5)
IN_(sp)_ + OUT_(w)__ST	3.0 (2.5 to 4.0)
OUT_(w)__LO	14.5 (14.0 to 14.5)

**Table 2:** Bacterial colony count on the chest area of scrub suits in each group. Values are median (interquartile range, IQR). *p*-values were calculated by using the Mann–Whitney U test in comparison with the control group, and their effect sizes, *r*, were also provided.

Group	Bacterial Colony Count (CFU), Median (IQR)	*p*-Value	Effect Size, *r*
Control	0.0 (0.0 to 1.0)	N/A	N/A
IN_(sp)__ST	1.0 (0.0 to 1.3)	0.142	0.35
IN_MID	0.5 (0.0 to 2.0)	0.348	0.21
IN_LO	2.0 (0.3 to 2.0)	0.019 *	0.51 ^†^
IN_(sp)_ + OUT_(w)__ST	5.0 (1.8 to 7.3)	<0.0001 *	0.73 ^†^
OUT_(w)__LO	14.5 (11.3 to 29.8)	<0.0001 *	0.85 ^†^

N/A: not available; *: *p* < 0.05; †: large effect size.

**Table 3:** Bacterial colony genus and species detected on the chest area of scrub suits in each group.

Group	Bacterial Colony Species (the Number of Cases Detected)
Control (*n* = 10)	GPR (2), *Bacillus subtilis* (1), *Micrococcus luteus* (1)
IN_(sp)__ST (*n* = 12)	*Bacillus subtilis* (3), *Bacillus cereus* (2), *Bacillus* species (1), *Staphylococcus hominis* * (1), *Micrococcus luteus* (1)
IN_MID (*n* = 12)	*Bacillus subtilis* (4), *Micrococcus luteus* (2), *Staphylococcus epidermidis* * (1), CNS * (1), *Bacillus megaterium* (1), *Sporosarcina koreensis* (1)
IN_LO (*n* = 11)	*Bacillus subtilis* (4), *Micrococcus luteus* (3), *Staphylococcus epidermidis* * (1), *Staphylococcus capitis* * (1), *Bacillus cereus* (1), *Bacillus megaterium* (1), *Roseomonas mucosa* (1), *Moraxella osloensis* (1)
IN_(sp)_ + OUT_(w)__ST (*n* = 20)	*Bacillus subtilis* (11), *Micrococcus luteus* (8), *Bacillus* species (3), *Acinetobacter* species (2), *Micrococcus* species (2), *Staphylococcus hominis* * (2), *Staphylococcus lugdunensis* * (1), *Staphylococcus saprophyticus* * (1), *Bacillus flexus* (1), *Bacillus cereus* (1), *Roseomonas mucosa* (1), *Kocuria* species (1), *Streptococcus* species (1)
OUT_(w)__LO (*n* = 10)	*Bacillus subtilis* (9), *Micrococcus luteus* (4), *Staphylococcus epidermidis* * (3), *Staphylococcus saprophyticus* * (2), *Staphylococcus hominis* * (1), *Aerococcus viridans* (1), *Enterococcus faecalis* (1), *Roseomonas mucosa* (1), *Pantoea septica* (1)

GPR: gram positive rod; *: coagulase negative staphylococci (CNS).

**Table 4:** Probability of contamination caused by any bacteria and CNS on the chest area of scrub suits in each group. *p*-values were calculated by using the chi-squared test in comparison with the control group, and their effect sizes, *φ*, were also provided.

Group	Any Bacterial Contamination			CNS Contamination		
	Probability (%)	*p*-Value	Effect Size, *φ*	Probability (%)	*p*-Value	Effect Size, *φ*
Control (*n* = 10)	40.0 (4/10)	N/A	N/A	0 (0/10)	N/A	N/A
IN_(sp)__ST (*n* = 12)	66.7 (8/12)	0.211	0.27	8.3 (1/12)	0.350	0.20
IN_MID (*n* = 12)	66.7 (8/12)	0.211	0.27	16.7 (2/12)	0.176	0.29
IN_LO (*n* = 11)	72.7 (8/11)	0.130	0.32	18.2 (2/11)	0.156	0.31
IN_(sp)_ + OUT_(w)__ST (*n* = 20)	100 (20/20)	0.0001 *	0.71 ^†^	20 (4/20)	0.129	0.28
OUT_(w)__LO (*n* = 10)	100 (10/10)	0.003 *	0.66 ^†^	50 (5/10)	0.010 *	0.58 ^†^

N/A: not available; *: *p* < 0.05; †: large effect size.

## Data Availability

Not applicable.
